# Strengthening medical education through health policy and management training: a cross-sectional study among Portuguese medical students

**DOI:** 10.3389/fpubh.2026.1726073

**Published:** 2026-03-04

**Authors:** Francisco Goiana-Da-Silva, Duarte Tude Graça, Miguel Peliteiro, Juliana Sá, Tiago Martins, Joana Praia, Inês Guerreiro, Alexandre Alves Rodrigues, Mario Amorim Lopes, Francisco Sousa Vieira, Tomás Pessoa-e-Costa, Fernando Correia, Alexandre Lourenço, Eduardo Costa, Alexandre Morais Nunes, Alexandra Bento, João Breda, Conceição Calhau, Jaime Branco, Maria Amélia Ferreira, Hutan Ashrafian, Maria Do Céu Machado, Ara Darzi

**Affiliations:** 1 Centre for Health Policy, Institute of Global Health Innovation, Imperial College London, London, United Kingdom; 2 NOVA Medical School, Universidade NOVA de Lisboa, Lisbon, Portugal; 3 Faculdade de Ciências da Saúde, Universidade da Beira Interior, Covilhã, Portugal; 4 Sword Health, Inc., Porto, Portugal; 5 Sword Health, Inc., Draper, UT, United States; 6 Faculdade de Medicina, Universidade de Lisboa, Lisbon, Portugal; 7 Centre for Public Administration and Public Policies, Institute of Social and Political Sciences, University of Lisbon, Lisbon, Portugal; 8 Well Partners Unipessoal LDA, Lisbon, Portugal; 9 INESC-TEC, Faculty of Engineering, University of Porto, Porto, Portugal; 10 Faculdade de Medicina, Universidade do Porto, Porto, Portugal; 11 Department of Dermatology, ULS Amadora Sintra, Lisbon, Portugal; 12 Department of Neurology, ULS Santo António, Porto, Portugal; 13 National School of Public Health (NOVA NSPH), Universidade NOVA de Lisboa, Lisbon, Portugal; 14 ULS Coimbra, Coimbra, Portugal; 15 Centre for Management Studies (CEGIST), Instituto Superior Técnico, Universidade de Lisboa, Lisbon, Portugal; 16 WHO Athens Quality of Care and Patient Safety Office, World Health Organization Regional Office for Europe, Athens, Greece; 17 Department of Nutrition and Metabolism, NOVA Medical School, Lisboa, Portugal; 18 Center for Health Technology Services Research (CINTESIS), Porto, Portugal; 19 Faculty of Medicine, Department of Surgery and Cancer, Imperial College London, London, United Kingdom

**Keywords:** curriculum development, governance, health policy, medical education, Portugal

## Abstract

**Background and objectives:**

Modern health systems require physicians to not only provide high-quality clinical care but also understand, navigate, and lead complex healthcare organizations. However, undergraduate medical education in Portugal remains predominantly focused on clinical skills, with minimal exposure to health policy and management (HPM). This study aimed to assess Portuguese medical students’ exposure to, attitudes toward, and preferences for HPM education.

**Methods:**

We conducted a cross-sectional survey of 483 medical students across 10 Portuguese medical schools. The questionnaire assessed prior exposure to HPM education, self-perceived knowledge of the national health system, curricular preferences, and civic participation.

**Results:**

Only 29.2% of participants (*n* = 141; 95% CI 25.1–33.2) reported any previous HPM training, and those with exposure were more likely to demonstrate higher institutional literacy and greater confidence in understanding health system governance. Overall, 94.8% supported the inclusion of HPM in the medical curriculum, and 64.4% supported making it compulsory, with stronger support among civically engaged students.

**Conclusion:**

Portuguese medical students had limited formal exposure to HPM but expressed strong demand for structured training in this area. These findings highlight a misalignment between current curricula and students’ perceived needs and support the introduction of a mandatory HPM course in Portuguese medical schools to better prepare future physicians for leadership and governance roles within the health system.

## Introduction

Modern healthcare systems are defined by their unprecedented intensity, persistent fiscal pressures, and greater political attention. Physicians are now responsible not only for delivering safe and effective clinical care but also for understanding, navigating, and shaping the systems in which care is delivered. Therefore, to provide strong, equitable, and sustainable health systems, physicians must be able to govern, lead, and interpret policy (1–3).

The World Health Organization has underscored the need for incorporating health policy and management into medical education to prepare professionals for the needs of the 21st century (4–7). Similarly, the Organisation for Economic Co-operation and Development (OECD) has highlighted the importance of building leadership and policy capacities as the foundation for ensuring high-performing health systems (8). These recommendations align with the influential Lancet Commission on Health Professionals for the 21st Century, which asserted that scientific competence alone is not sufficient; health professionals must also be empowered to operate as leaders, advocates, and institutional actors (3, 5, 9).

In fact, several countries have already begun restructuring their curricula as a consequence. In the UK, the Royal Colleges and the General Medical Council have committed to the embedding of leadership, management, and health policy education within undergraduate and postgraduate training. In Canada, the CanMEDS system unequivocally embeds the “Leader” and “Health Advocate” roles as core to the development of their professional identity. Complementary reforms have also been observed in Nordic countries and other parts of the European continent, where systems thinking and leadership are fast emerging as core competencies rather than electives (10–13). Collectively, these directions mirror a global convergence on the idea that HPM education has to be a part of professional formation (14–16).

In Portugal, analyses of the national curriculum across all medical schools confirm that, despite recognizing the civic role of physicians for several decades, medical education continues to prioritize clinical expertise over system-level competencies (17–27). This gap is particularly concerning given the ongoing transformation of the National Health Service (SNS), which is currently facing financial strain, organizational restructuring, and technological changes (28).

Evidence that change is indeed on the horizon is present. The Council of Portuguese Medical Schools has argued that all graduates must achieve an appreciation of several types of health systems as well as how the public, private, and social sectors engage (29). Similarly, the Portuguese Agency for Evaluation and Accreditation of Higher Education has called for enhanced integration of health management, governance, and organizational competencies to ensure that medical education responds to national priorities (30).

Medical students play an equally essential part in reform efforts. As immediate stakeholders and future professionals, students can provide first-hand feedback on whether training is sufficient and relevant. Best practices in medical education emphasize the importance of incorporating students’ contributions to ensure reforms reflect professional realities, assist identity formation, and improve institutional responsiveness (31, 32).

Despite these national and international calls for change, there is currently no nationally representative data on how Portuguese medical students experience and perceive HPM. Specifically, it is unknown to what extent students are exposed to HPM content during their training, how prepared they feel to understand system governance, and what their preferences are regarding the format, timing, and mandatory nature of HPM training. This lack of empirical data limits the capacity of curriculum designers and medical schools to plan reforms that are responsive both to system needs and to students’ expectations. This study addresses the research gap by surveying medical students across Portuguese medical schools to: (i) describe prior exposure to HPM education, (ii) assess self-perceived knowledge of the national health system, and (iii) explore preferences and attitudes regarding the inclusion of HPM in undergraduate curricula. By providing nationally representative data on these dimensions, the study aims to inform curriculum development on how to integrate HPM training into Portuguese medical education.

## Methods

### Study design and setting

This was a cross-sectional, web-based survey of medical students enrolled in Portuguese medical schools during the 2024/2025 academic year (years 1–6). The survey was administered using Google Forms® and was open between 21 May and 5 July 2025. Eligible participants were students aged 18 years or older enrolled in an accredited undergraduate medical degree program in Portugal. Participants were unpaid, voluntary, and anonymous, and electronic informed consent was obtained before accessing the questionnaire.

### Target population and sampling

The questionnaire was self-administered and distributed via institutional mailing lists, student associations, and social media channels, complemented by direct contact with student representatives to maximize reach across schools and academic years. A total of 545 responses were received during the data collection period. After applying predefined inclusion and exclusion criteria (age ≥18 years, enrolment in an accredited medical program in Portugal, and registration in years 1–6) and removing invalid or substantially incomplete questionnaires, 483 valid responses remained, corresponding to 88.6% of all submissions. The final sample included students from 10 of the 11 accredited Portuguese medical schools and covered all academic years. *A priori* sample size calculation, assuming a 95% confidence level, 5% margin of error, and an anticipated response proportion of 50%, indicated a minimum required sample of 372 respondents; the final sample of 483 students exceeded this threshold. The total number of eligible medical students in Portugal was used to estimate the survey coverage and the overall response rate. As the survey was anonymous and voluntary, reasons for non-response could not be ascertained, and the possibility of selection bias toward more motivated or civically engaged students is acknowledged in the Limitations section.

### Questionnaire development and content

The questionnaire was adapted from a previously validated questionnaire by Malik et al. (33), which explored medical students’ exposure to and perceptions of health policy education, and was tailored to the Portuguese context through expert consultation and cognitive pre-testing with 10 medical students (33).

Building on this study, the questionnaire was translated and culturally adapted to the Portuguese context through expert consultation and cognitive pre-testing with medical students. Content validity of the adapted version was assessed by an expert panel comprising co-authors of this study with extensive experience in health policy and medical education, who independently reviewed all items for relevance, clarity, and cultural appropriateness, and discrepancies were resolved through iterative discussion until consensus was reached. This panel included eight experts in health policy and medical education from six different faculties across four universities.

The instrument included the following domains: sociodemographic characteristics (age, gender identity, academic year, and medical school); exposure to health policy and management training (type, duration, compulsory vs. elective, and perceived adequacy); knowledge and awareness of the Portuguese National Health Service (SNS) and its governance structures; civic engagement (membership in student or professional organizations, participation in advocacy); and preferences regarding the inclusion, format, timing, and nature of health policy and management training in the undergraduate curriculum. Self-perceived knowledge of the SNS and health system governance was assessed on a 6-point Likert scale from 0 (“no knowledge at all”) to 5 (“very high knowledge”).

### Data management and analysis

Data were exported from Google Forms® into Microsoft Excel®. Cases were screened for eligibility (age, degree program, and academic year) and completeness; responses that did not meet inclusion criteria or were substantially incomplete were excluded. Descriptive statistics (frequencies, percentages, means, and standard deviations, as appropriate) were used to summarize participant characteristics and key survey variables. Bivariate associations between categorical variables (e.g., prior HPM training, civic engagement, and support for mandatory courses) were examined using Pearson’s chi-square tests, with a significance level of *p* < 0.05.

### Ethical considerations

Before initiating the study, the authors consulted the President of the Nova Medical School Ethics Research Committee to determine the need for formal ethics approval. The Committee issued a waiver (reference 140/2025/CEFCM), stating that full review was not required because the project consisted of a pedagogical/quality-improvement activity using an anonymous, voluntary survey of adult students, without collecting personal identifiers or sensitive data and with no foreseeable risks to participants. All students received information about the study objectives and data protection procedures and provided electronic informed consent, with the option to withdraw at any point before submitting their responses.

## Results

### Respondent characteristics

A total of 483 valid responses were analyzed, representing students from 10 of the 11 accredited medical schools in Portugal. All academic years were included, with 8.3% of respondents enrolled in the first year, 10.4% in the second year, 15.7% in the third year, 20.7% in the fourth year, 17.8% in the fifth year, and 27.1% in the sixth year. The Faculty of Medicine at the University of Lisbon (32.9%) and NOVA Medical School (26.5%) accounted for the largest shares of respondents, together comprising approximately 60% of the total sample. Smaller yet significant contributions came from the Universities of Coimbra (9.9%) and Beira Interior (8.9%). The remaining schools each accounted for less than 7% of the responses (Table 1). The mean age of participants was 22.9 years (standard deviation 3.0; range 18–35 years), and the majority of respondents identified as women (71.0%), followed by men (28.2%) and a small minority identifying as non-binary or another gender (Table 2).

**Table 1:** Number and percentage of medical student respondents by academic year.

Academic year	*n* (%)
1st year	40 (8,3)
2nd year	50 (10,4)
3rd year	76 (15,7)
4th year	100 (20,7)
5th year	86 (17,8)
6th year	131 (27,1)
Total	483 (100,0)

**Table 2:** Socio-demographic characteristics of medical student respondents at baseline (*N* = 483).

Baseline characteristic	*n* ^b^	%
Age (years)		
Mean (SD)^a^	22.9 (3.0)	
Median (Range)	23 (18–35)	
Gender		
Female	343	71.0
Male	136	28.2
Non-binary	3	0.6
Other	1	0.2

Percentages are calculated out of *N* = 483.

^a^Mean and standard deviation are calculated using numeric ages from 18 to 35 years.

^b^Academic year values are aggregated across all participating medical schools and correspond to those reported in Table 1 of the manuscript.

### Prior exposure to health policy and management training

Only 29.2% (*n* = 141) of respondents reported having received any form of HPM training during medical school, most often as part of fragmented content within broader public health courses. Among those with prior exposure, training was frequently described as superficial and insufficient to develop practical skills in navigating health systems. Conversely, the majority (70.8%, *n* = 342) reported no prior structured education in this domain (Table 3).

**Table 3:** Number and percentage of medical students with and without prior HPM training, and proportion wanting training among those without prior exposure, by medical school.

Medical school	Had HPM Training *n* (%)	No HPM Training *n* (%)	Students without HPM training who would like to receive it, *n*/*N* (%)
Faculty of Medicine, University of Lisbon	47 (29.6)	112 (70.4)	104/112 (92.9)
NOVA Medical School	23 (18.0)	105 (82.0)	99/105 (94.3)
Faculty of Medicine, University of Coimbra	12 (25.0)	36 (75.0)	35/36 (97.2)
Faculty of Health Sciences, University of Beira Interior	34 (79.1)	9 (20.9)	9/9 (100)
Faculty of Medicine, University of Porto	6 (17.6)	28 (82.4)	26/28 (92.9)
Faculty of Medicine and Biomedical Sciences, University of Algarve	2 (7.1)	26 (92.9)	25/26 (96.2)
School of Medicine, University of Minho	8 (38.1)	13 (61.9)	13/13 (100)
Abel Salazar Institute of Biomedical Sciences (ICBAS), University of Porto	9 (69.2)	4 (30.8)	4/4 (100)
Department of Medical Sciences, University of Aveiro	0 (0)	7 (100)	7/7 (100)
Faculty of Medicine, Portuguese Catholic University	0 (0)	2 (100)	2/2 (100)

### Support for mandatory training

**Figure 1:**
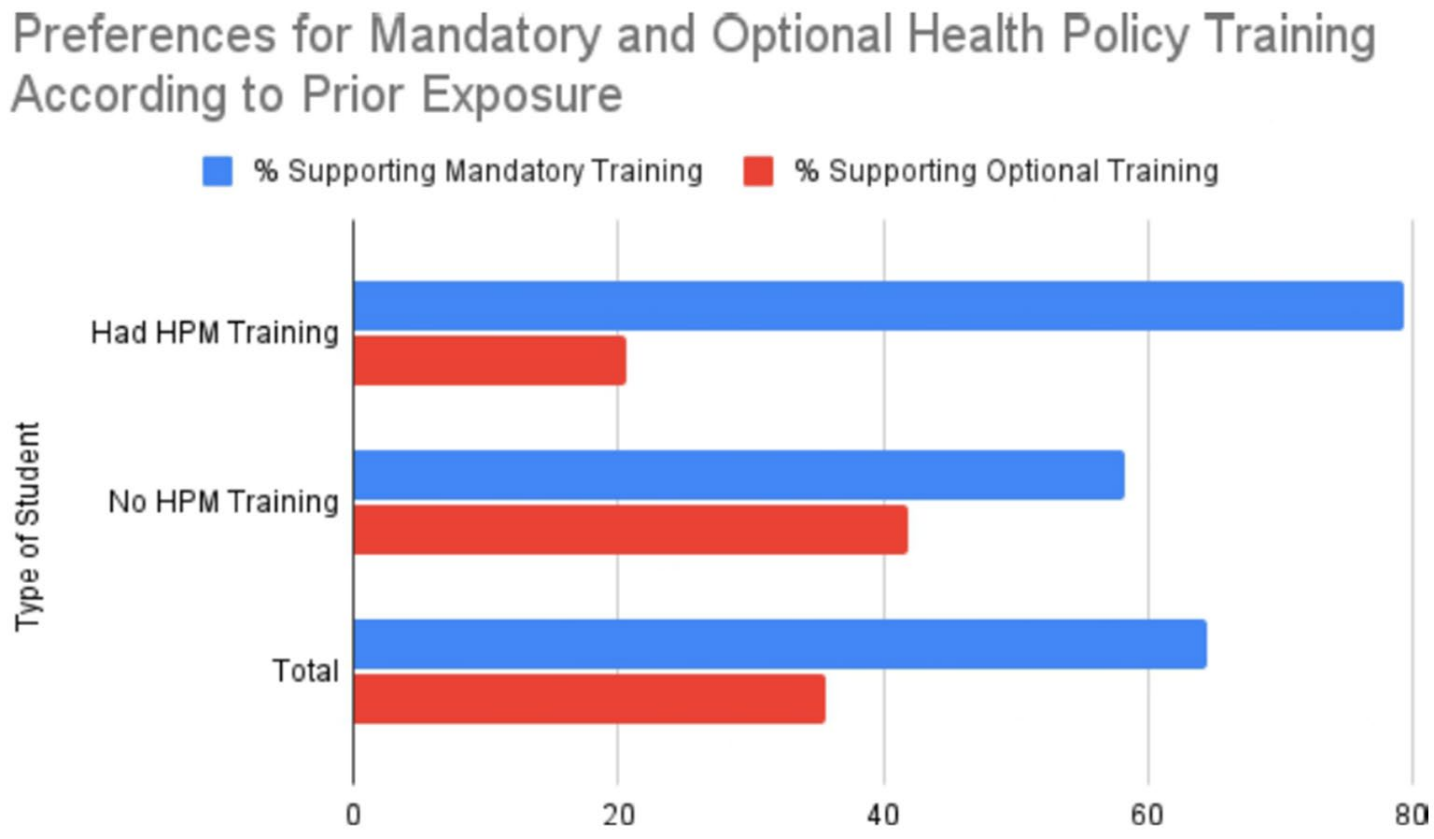
Support levels for health policy and management training by student group.

A total of 64.4% of students (*n* = 483), both with and without prior HPM training, expressed support for the creation of a mandatory course in this area (Table 4). Support was notably stronger (79.4%, *n* = 112) among students who had already experienced some form of structured HPM modules, compared with students without such training (58.2%, *n* = 199/342; *χ*^2^ = 18.7, *p* < 0.0001), suggesting that exposure to structured HPM modules reinforces recognition of their relevance (Figure 1).

**Table 4:** Student endorsement of HPM training and format preferences by training background.

Type of Student	Number of students	% supporting training	% supporting mandatory training	% supporting optional training
Had HPM Training	141	134	112	29
		(95.0%)	(79.4%)	(20.6%)
No HPM Training	342	324	199	143
		(94.7%)	(58.2%)	(41.8%)
Total	483	94.8%	64.4%	35.6%

Importantly, students without prior training still acknowledge the importance of HPM, although they often express difficulty articulating its core components or understanding its practical implications.

### Self-perceived institutional literacy

Students’ self-rated knowledge of the SNS and health system governance was generally low, with an overall mean of 2.5 on a 0–5 Likert scale. Those with prior HPM training reported significantly higher levels of knowledge (mean = 3.1) than their peers (mean = 2.3). While 55% of trained students rated their knowledge as high (≥4), only 27% of untrained students did so. Conversely, 31% of untrained students reported very low knowledge (≤2), compared to just 14% of trained students.

### Desire for additional training

**Figure 2:**
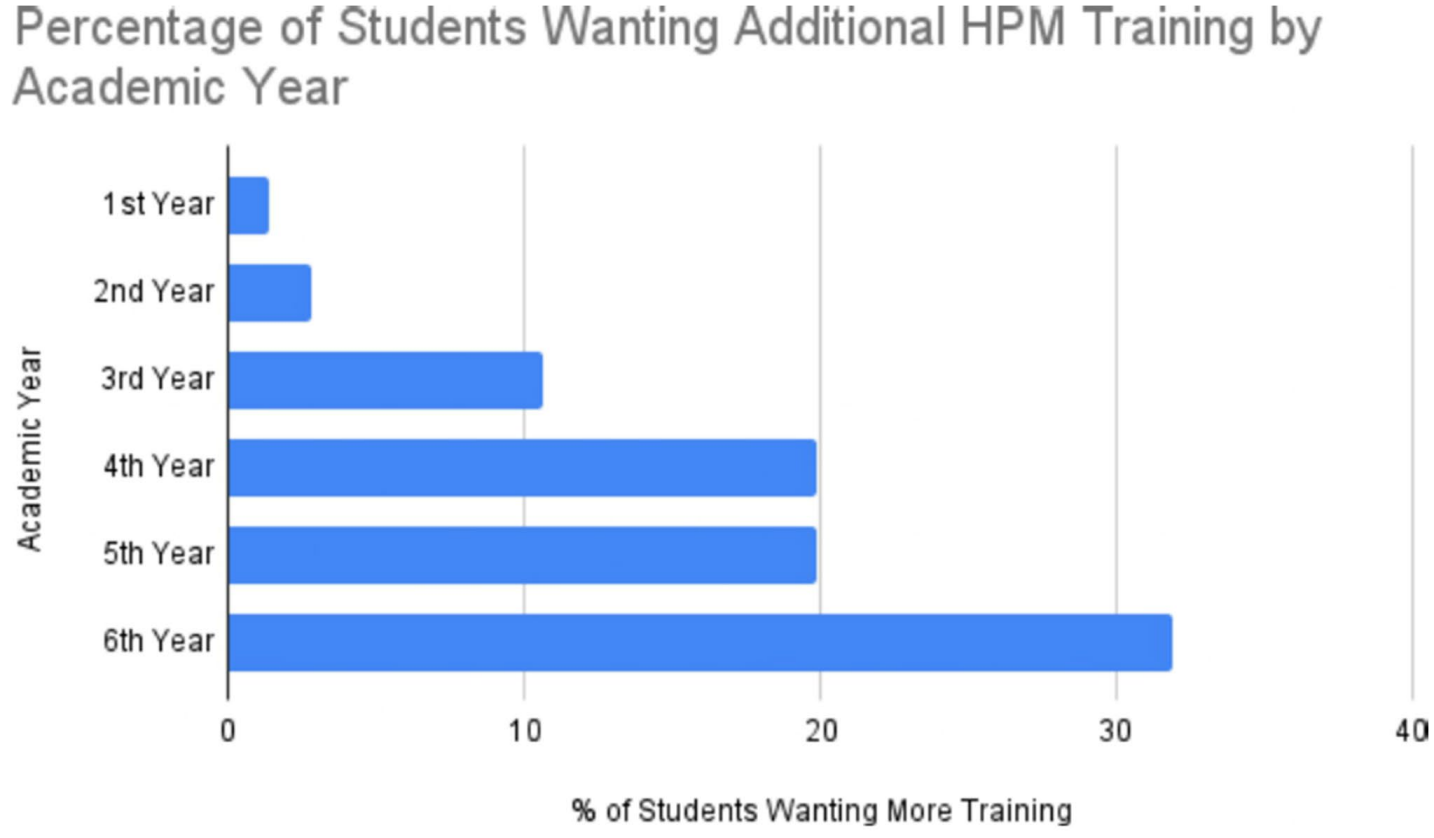
Percentage of previously trained students requesting additional HPM training by academic year.

Among students with prior HPM exposure, 86.5% expressed interest in receiving additional instruction. This demand was strongest among senior students, with 91.8% of 6th-year respondents and 73.7% of 5th-year respondents indicating a desire for further training, suggesting that clinical experience increases recognition of the importance of system-level competencies (Figure 2).

### Preferred curricular design and timing

Overall, 61.9% of respondents expressed a preference for HPM to be taught as a dedicated curricular unit, rather than being integrated into existing courses (Table 5). The majority of respondents favored introduction during the fourth (18.4%) or fifth (25.9%) years, aligning with the period of transition to clinical practice. Students suggested reallocating European Credit Transfer and Accumulation System credits from existing courses, most frequently from “Public Health” and “Introduction to Medicine,” to accommodate this addition without increasing the overall workload. Given that the majority of students preferred to place the HPM course in the fourth or fifth year and “Introduction to Medicine” is typically taught in the early years, the most feasible and widely supported option appears to be a partial redistribution of the workload currently allocated to “Public Health.” This would allow for the integration of HPM content without increasing the overall demands on students, while aligning with their preferred timing in the curriculum.

**Table 5:** Preferences regarding HPM curricular format, timing and priority topics among Portuguese medical students (*N* = 483).

Domain	Category	*n* (%)
Curricular format	Dedicated curricular unit	299 (61.9)
	Integrated into existing courses	169 (35.0)
	Other / No preference	15 (3.1)
Preferred timing	4th year	89 (18.4)
	5th year	125 (25.9)
	6th year	46 (9.5)
	Other curricular years	167 (34.6)
	No preference	56 (11.6)
Priority topics	Structure and functioning of the SNS	363 (75.1)
	Health system financing and sustainability	266 (55.2)
	Hospital leadership and management	201 (41.7)
	Health economics	156 (32.3)
	Public health and healthcare planning	131 (27.2)
	Health equity and ethics	109 (22.6)

Percentages are calculated out of *N* = 483.

### Priority topics

The most frequently identified priority topics are summarized in Table 5: (1) structure and functioning of the SNS (75.1%), (2) health system financing and sustainability (55.2%), (3) hospital leadership and management (41.7%), (4) health economics (32.3%), (5) public health and healthcare planning (27.2%), and (6) health equity and ethics (22.6%).

### Civic engagement

A statistically significant association was observed between civic engagement and support for mandatory HPM education (*χ*^2^ = 4.65, *p* = 0.031). Students who participated in associations or held extracurricular leadership roles were more likely to endorse compulsory training (70.7%, *n* = 184) compared to their non-engaged peers (60.5%, *n* = 299).

## Discussion

This national survey provides the first national description of the current situation of Portuguese medical students’ perceptions of the introduction of HPM into undergraduate medical education. The findings reveal a large gap in formal HPM education with a corresponding stated need for organized teaching, suggesting both an unmet education requirement and potential for enrichment of the curriculum.

### Key findings and interpretation

This nationally representative survey shows that, while 94.8% of Portuguese medical students supported the inclusion of health policy and management (HPM) in the medical curriculum, only 29.2% (*n* = 141; 95% CI 25.1–33.2) reported any prior exposure to it. Furthermore, 64.4% of students supported making HPM compulsory. This significant discrepancy between limited training and strong student demand aligns with national curriculum analysis indicating that clinical expertise is prioritized over HPM competencies, despite the financial pressures, organizational restructuring, and technological changes facing the Portuguese National Health Service. These findings align with international recommendations from the World Health Organization and the Organisation for Economic Co-operation and Development, which emphasize policy, leadership, and governance capabilities as core requirements for 21st-century health professionals (8). They also align with commentaries calling for health policy education to be reconsidered in medical schools (34).

A second key finding is that students with prior experience of HPM were more likely to report a higher level of institutional literacy and greater confidence in their understanding of health system governance compared to their peers without such experience. This suggests that relatively short or elective educational experiences can influence how students perceive decision-making structures, financing arrangements, and accountability mechanisms. Similar findings were reported by Malik et al. in their international study of health policy teaching in UK medical schools, which found that students who had received health policy education felt better prepared to engage with system-level issues. Subsequent analyses have argued for broadening and deepening such training in undergraduate curricula (34). Reviews of leadership and management training in medical education also demonstrate consistent improvements in knowledge, attitudes, and self-efficacy following structured interventions, which supports the plausibility of the mechanisms observed in our data (35). However, the predominantly optional and extracurricular nature of HPM opportunities in Portugal likely restricts their reach, potentially reinforcing inequities between students who can access such experiences and those who cannot.

A third noteworthy outcome is that students who are civically engaged, that is, those who reported participating in student associations, advocacy initiatives, or representative bodies, were more likely to endorse compulsory HPM training compared to their less engaged peers. This suggests that students already involved in institutional or civic structures recognize the importance of policy, governance, and management competencies for their future professional roles. This finding is consistent with competency frameworks such as CanMEDS, which explicitly define the “Leader” and “Health Advocate” roles as fundamental to medical professionalism. It also aligns with international discussions on how the clinical learning environment can better support these roles (10–13).

Another key observation is that the overall pattern of low formal exposure, strong perceived relevance, and substantial support for compulsory HPM training mirrors international trends in medical education. Studies from the United Kingdom, Canada, the Nordic countries, and other European settings have documented the progressive integration of leadership, management, and health policy content into undergraduate curricula (6, 33, 35–37). This integration is often guided by national competence frameworks and regulatory standards. These studies also report that students appreciate these components as valuable preparation for their future roles in system improvement and governance (34). By utilizing an adapted version of a previously validated questionnaire on medical students’ involvement in and attitudes toward health policy, this study has made cautious comparisons of exposure, attitudes, and preferences across different contexts, highlighting a broader convergence on the importance of structured HPM training.

### Limitations

This study has certain limitations that should be taken into account when interpreting the findings. First, the cross-sectional design does not allow for causal inferences to be made about the relationship between exposure to HPM training, institutional literacy, and attitudes toward curricular reform. Second, participation was based on a voluntary web survey, which may have introduced selection bias by over-representing more motivated or civically engaged students. Although the sample included students from 10 out of the 11 accredited Portuguese medical schools and all academic years, the distribution of respondents was highly uneven across institutions, with almost 60% of participants coming from just two faculties; consequently, under-represented schools may not be adequately reflected, and the generalizability of the findings to all medical schools should be considered with caution. Third, as all variables were self-reported, there is a possibility of recall and social desirability bias. However, anonymity and careful item wording were used to mitigate these effects. Finally, as the study focused on students’ perceptions and self-perceived knowledge rather than objective measures of competence or longitudinal outcomes, future research should complement these results with evaluative designs that assess the impact of specific HPM curricular interventions over time.

## Conclusion

This study shows that fewer than one-third of Portuguese medical students have received any formal HPM training, while more than 9 in 10 support its inclusion in the curriculum and almost two-thirds favor making it compulsory. The observed associations between HPM exposure and higher institutional literacy, greater confidence in understanding health system governance, and increased levels of civic engagement suggest that integrating structured HPM teaching into undergraduate medical education could strengthen the leadership, advocacy, and governance skills necessary for the future sustainability and effectiveness of the Portuguese National Health Service.

These findings provide a clear direction for educators and curriculum committees to design and implement core HPM modules with explicit learning outcomes, clear links to clinical practice, and active, experiential pedagogical approaches that expose students to real-world governance and management challenges.

Future research should include longitudinal evaluations of newly implemented HPM curricula, assessing their impact on students’ knowledge, attitudes, career trajectories, and subsequent involvement in governance and leadership roles. Complementary qualitative studies involving students, faculty members, managers, and policymakers could further clarify the barriers to, and facilitators of, the integration of HPM in medical education and inform context-sensitive implementation models. Together, the present findings and future studies could support a coherent reform agenda in which Portuguese medical education prepares physicians to be competent clinicians and informed participants in health system governance and policy.

## Data Availability

The original contributions presented in the study are included in the article/Supplementary material, further inquiries can be directed to the corresponding authors.
