# Supplementary material for: Strengthening medical education through health policy and management training: a cross-sectional study among Portuguese medical students
Source: Front Public Health. 2026 Mar 4;14:1726073. doi: 10.3389/fpubh.2026.1726073 (PMC12996082; doi:10.3389/fpubh.2026.1726073)
Supplement: Supplementary file 1 [file Data_Sheet_1.pdf]

Pedagogical Evaluation and Curricular Development Survey on Health Policy and Management

SCOPE

This questionnaire is addressed to medical students undergoing their training in Portugal and aims to collect perceptions regarding the potential creation of mandatory curricular units dedicated to Health Policy and Management. It seeks to understand the current level of knowledge, educational experiences, opinions, and suggestions for the curricular integration of these topics.

CONFIDENTIALITY

By completing this questionnaire, I confirm that I agree for my responses to be included anonymously in the final report and that the anonymized data may be confidentially stored for a period of 6 years.

KEY DEFINITIONS

Health Policy: Refers to the decisions, plans, and actions taken to achieve specific health objectives within a society. It defines a vision for the future, establishes goals, priorities, the roles of different groups, and promotes consensus.

Health Management: The organization, coordination, and supervision of health resources and services (public and/or private) with the aim of ensuring their efficient, sustainable, and equitable functioning.

PART I: Sample Characterization

1. What is your age?

Mark only one option.

- 17 years
- 18 years
- 19 years
- 20 years
- 21 years

- 22 years
- 23 years
- 24 years
- 25 years
- 26 years
- 27 years
- 28 years
- 29 years
- 30 years
- 31 years
- 32 years
- 33 years
- 34 years
- 35 years
- 35 years

2. What is your gender?

Mark only one option.

- Female

- Male
- Non-binary
- Other

3. Which medical school do you attend?
Mark only one option.

- Department of Medical Sciences, University of Aveiro
- School of Medicine, University of Minho
- School of Medicine and Biomedical Sciences, Universidade Fernando Pessoa
- Faculty of Health Sciences, University of Beira Interior
- Faculty of Medicine, University of Porto
- Faculty of Medicine, University of Coimbra
- Faculty of Medicine, Portuguese Catholic University
- Faculty of Medicine, University of Lisbon
- Faculty of Medicine and Biomedical Sciences, University of Algarve
- Abel Salazar Institute of Biomedical Sciences, University of Porto
- Nova Medical School | Faculty of Medical Sciences, Universidade Nova de Lisboa

4. What is your year of study / training level?
Mark only one option.

- 1st year
- 2nd year
- 3rd year
- 4th year
- 5th year
- 6th year

5. In your opinion, what is the impact of public policies on the health of individuals and societies? *(Likert-type scale)*

- 0 – None
- 1 – Very low
- 2 – Low
- 3 – Moderate
- 4 – High
- 5 – Very high

PART II: Previous Training in Health Policy and Management

6. Have you received any training in Health Policy and/or Management during your medical course?
Mark only one option.

- Yes (Skip to question 19)

- No (Continue to question 7)

PART III A: Teaching of Health Policy and Management (No HPM Training)

7. What is your current level of knowledge about health management/policy? *(Likert-type scale)*

8. What is your current level of knowledge about the organization and functioning of National Health Service institutions? *(Likert-type scale)*

9. Would you have liked to receive training in health policy and management during your medical course?
Mark only one option.

- Yes
- No

10. Do you think medical students' training in health management and policy should be:
Mark only one option.

- Mandatory
- Optional

11. In a context of mandatory training in management and policy during the medical course, which format would you consider most appropriate?
Mark only one option.

- Integrated topics within the programs of existing courses
- As a standalone course
- Other (please specify):

12. In a context of optional training in management and policy during the medical course, which format would you consider most appropriate?
Mark only one option.

- As a standalone optional course
- As a credited extracurricular activity (intensive course, seminar, workshop)
- Other (please specify):

13. If a new mandatory course focused on Health Policy and Management were introduced in your medical school, in which year of the course would it be most appropriate?
Mark only one option.

- 1st year
- 2nd year
- 3rd year
- 4th year
- 5th year
- 6th year
- No preference

14. Which topics would you consider a priority to include in this new course? *(Check all that apply)*

- Organization/Functioning of the National Health Service
- Financing and sustainability of health systems
- Leadership and hospital management

- Public health and health planning
- Health economics
- Health technology assessment
- Health literacy
- Ethics and equity in health policy
- Planning and strategy
- Other (please specify):

15. If health management and policy topics were integrated as discrete elements within another existing mandatory course, which course would seem most appropriate?

Mark only one option.

- Course currently teaching Public Health topics
- Course currently teaching Introduction to Medicine topics
- Course currently teaching General and Family Medicine topics
- Course currently teaching Ethics and Professionalism topics
- Other (please specify):

16. If the creation of a new mandatory course in Health Policy and Management required reducing the workload of another existing course, which course would that be?

Mark only one option.

- Course currently teaching Public Health topics

- Course currently teaching Introduction to Medicine topics
- Course currently teaching General and Family Medicine topics
- Course currently teaching Ethics and Professionalism topics
- Other (please specify):

17. If a new standalone and mandatory course in health management and policy were to be created, it should be implemented:
Mark only one option.

- By increasing the total course workload
- By reducing content in existing mandatory courses to create space for a new individual course while keeping the total workload unchanged
- Other (please specify):

18. What would be the appropriate total workload/number of classes for a new course?
Mark only one option.

- 5 classes of 2 hours
- 10 classes of 2 hours
- 15 classes of 2 hours
- 20 classes of 2 hours

PART III B: Teaching of Health Policy and Management (Had HPM Training)

19. What is your current level of knowledge about health management/policy? *(Likert-type scale)*

20. What is your current level of knowledge about the organization and functioning of National Health Service institutions? *(Likert-type scale)*

21. In what format did you receive training in health management and policy during your medical course?
Mark only one option.

- Discrete topics integrated into existing course programs
- As a standalone mandatory course
- As a standalone optional course
- As a credited extracurricular activity
- Other (please specify):

22. Would you like / have liked to receive more training in health policy and management during your medical course?
Mark only one option.

- Yes
- No

23. Do you think all medical students should receive training in health management and policy?
Mark only one option.

- Yes
- No

24. Do you think medical students' training in health management and policy should be:
Mark only one option.

- Mandatory
- Optional

25. In a context of mandatory training in management and policy, which format would you consider most appropriate?
Mark only one option.

- Discrete topics integrated into existing course programs
- As a standalone course
- Other (please specify):

26. In a context of optional training in management and policy, which format would you consider most appropriate?
Mark only one option.

- As a standalone optional course
- As a credited extracurricular activity
- Other (please specify):

27. If a new mandatory course focused on Health Policy and Management were introduced in any medical school, in which year of the course would it be most appropriate?
Mark only one option.

- 1st year
- 2nd year
- 3rd year
- 4th year

- 5th year
- 6th year
- No preference

28. If the creation of a new mandatory course in Health Policy and Management required reducing the workload of another existing course, which course would that be?
Mark only one option.

- Course currently teaching Public Health topics
- Course currently teaching Introduction to Medicine topics
- Course currently teaching General and Family Medicine topics
- Course currently teaching Ethics and Professionalism topics
- Other (please specify):

29. Which topics would you consider a priority to include in this new course? *(Check all that apply)*

- Organization/Functioning of the National Health Service
- Financing and sustainability of health systems
- Leadership and hospital management
- Public health and health planning
- Health economics
- Health technology assessment

- Health literacy
- Ethics and equity in health policy
- Planning and strategy
- Other (please specify):

30. If health management and policy topics were integrated as discrete elements within another existing mandatory course, which course would seem most appropriate?

Mark only one option.

- Course currently teaching Public Health topics
- Course currently teaching Introduction to Medicine topics
- Course currently teaching General and Family Medicine topics
- Course currently teaching Ethics and Professionalism topics
- Other (please specify):

31. If a new standalone and mandatory course in health management and policy were to be created, it should be implemented:

Mark only one option.

- By increasing the total course workload
- By reducing content in existing mandatory courses to create space for a new individual course while keeping the total workload unchanged
- Other (please specify):

32. What would be the appropriate total workload/number of classes for a new

course?

Mark only one option.

- 5 classes of 2 hours
- 10 classes of 2 hours
- 15 classes of 2 hours
- 20 classes of 2 hours

PART IV: Involvement and Future

33. Have you ever been or are you currently involved in student associations/representation or activities related to health policy?

Mark only one option.

- Yes
- No

34. Would you consider working on topics or in institutions related to health governance/policy in your future career?

Mark only one option.

- Yes
- No

35. Do you currently consider that you have an adequate/sufficient level of knowledge about the structure and functioning model of the National Health Service to join one of its healthcare provider institutions as a paid professional worker?

Mark only one option.

- Yes

- No
